# Effects of *Euphorbia humifusa* extract on growth performance and serum biomarkers in preweaned calves

**DOI:** 10.3389/fvets.2025.1631972

**Published:** 2025-11-20

**Authors:** Chuntao Zhang, Zhongying Xing, Guishan Xu, Yan Tu, Qiyu Diao

**Affiliations:** 1 Key Laboratory of Feed Biotechnology of Ministry of Agriculture and Rural Affairs, Feed Research Institute of Chinese Academy of Agricultural Sciences, Beijing, China; 2 ‌Animal Science and Technology College, Tarim University, Alar, China

**Keywords:** antioxidant, calves growth, *Euphorbia humifusa* extract, milk replacer, network pharmacology, plant extract

## Abstract

**Introduction:**

The juvenile period represents a critical rearing phase in animals, during which rearing quality directly impacts adult productive performance. Plant extracts have been used as feed additives to promote growth, inhibit bacteria, enhance immunity, improve animal health, and ensure the safety of animal products. Therefore, our study aimed to investigate the effects of *Euphorbia humifusa* extract (EHE) on growth performance, serum biomarkers and antioxidant mechanisms in preweaning calves.

**Methods:**

Forty-eight newborn calves were randomly allocated to four groups (12 calves/group) and fed milk replacer supplemented with 0 mg (control, CON), 400 mg (Group A), 800 mg (Group B), or 1,200 mg (Group C) of EHE. Body weight and serum biomarkers were measured on d 30 and 60. Network pharmacology was employed to identify EHE-related antioxidant targets, followed by Gene Ontology (GO) and Kyoto Encyclopedia of Genes and Genomes (KEGG) enrichment analyses.

**Results:**

Calves in group C exhibited significantly higher average daily gain (ADG) compared with CON during d 30–60. Both dry matter intake (DMI) and ADG across treatment groups demonstrated a dose-dependent increase. Serum growth hormone (GH) shows the same trend as daily weight gain and feed intake. Serum analysis revealed that superoxide dismutase (SOD) activity in group C was significantly elevated versus CON, Network pharmacology identified 150 potential antioxidant targets of EHE, primarily enriched in pathways associated with cancer, hepatic injury, apoptosis, and viral infection, suggesting immune-modulatory effects.

**Discussion:**

Based on these findings, it can be inferred that supplementing milk replacer with EHE enhances calf growth performance, regulating oxidative stress, and it regulates signaling pathways related to immune response and apoptosis through interactions with key targets such as IL6, TP53, MAPK1, AKT1, TNF, BCL2, and ESR1.

## Introduction

During the lactation period, calves have not yet fully developed physiological functions and experience significant changes after being separated from their mothers. This stage exposes calves to multiple challenges, including birth, environmental and temperature changes, which easily trigger stress responses and subsequently affect their growth, development, and health. As the most vulnerable phase in the entire lifecycle of dairy cows, the quality of rearing during this period directly influences the productive performance of adult cows and is closely related to the safety and quality of subsequent animal products (1).

Antibiotics, when used as feed additives in calf diets, have historically played roles in disease resistance and growth promotion. However, the widespread use of antibiotics has increasingly revealed drawbacks. Against the backdrop of heightened public health awareness, food safety has become a critical societal issue, and China’s livestock industry is accelerating its transition to a green, intensive, and high-quality development model. Natural product extracts, as feed additives, offer benefits such as promoting growth, inhibiting bacteria, enhancing immunity, improving animal health, and ensuring the safety of animal products (2).

*Euphorbia humifusa* (dried whole herb of *Euphorbia humifusa* or *Euphorbia maculata* in the Euphorbiaceae family), also known as Caoxuejie, Xuejianchoucao, or Tiexiancao, is rich in bioactive compounds and exhibits broad pharmacological activities. It is a commonly used herb in Mongolian and Uyghur medicine and widely applied in traditional Chinese medicine. The major components of EHE include flavonoids such as quercetin, kaempferol, rutin, luteolin, and apigenin—polyphenolic compounds that mediate diverse biological effects, including antioxidant, antitumor, immune-enhancing, and antibacterial activities (3). Clinically, it is used to clear heat and detoxify, treat boils, dysentery, enteritis, hemoptysis, and bloody stools (4).

Our research team has over 20 years of experience in the field of calves, and previous studies have shown that, dietary supplementation with plant extracts containing flavonoids (e.g., baicalin) and polyphenols (e.g., rutin, pulchinenoside B4, resveratrol) improves growth performance in preweaned calves and reduces the incidence and severity of preweaning diarrhea (5–7). These extracts also enhance lactation performance in heat-stressed (8, 9) and lactating dairy cows (10), improve body weight, average daily gain (ADG), average daily feed intake (ADFI), and feed conversion ratio in broilers, and decrease disease incidence (11). *Euphorbia humifusa* contains multiple bioactive components that promote growth and physiological health. Zhang et al. (12) reported that EHE intervention in ulcerative colitis mice reduced Disease Activity Index (DAI) scores, body weight loss, colonic ulcers, and intestinal epithelial barrier damage, while downregulating inflammatory cytokine expression (Experimental study on EHE improving ulcerative colitis in mice via miR-124-3p/ELF3-mediated NLRP3 regulation). The experimental results of other animals also showed that extracts from similar plants have strong free radical scavenging and inhibitory effects, and their antioxidant capacity is ranked in the following order: *Euphorbia humifusa* > Scabiosa comosa > Cymbaria daurica > *Choerospondias axillaris* > Oxytropis myriophylla. Based on the types and contents of active ingredients in EHE (Table 1), we believe that EHE has the potential to improve the production performance and health of livestock. However, its application in calf production remains unstudied. Therefore, this study aimed to investigate the effects of EHE on growth performance, serum biomarkers, and antioxidant mechanisms in preweaning calves through animal feeding trials and network pharmacology, to evaluate its potential application and provide a scientific basis for its use in animal husbandry.

**Table 1:** List of active ingredients in EHE.

Items	Ingredients	Contents
Saponin	Glycoside compounds	11.00, %
Total Flavonoids	Flavonoids	3.23, mg/g
saccharides	Maltose	22.49, ug/mg
	Glucose	12.66, ug/mg
	Fructose	5.69, ug/mg
	L-Fucose	2.53, ug/mg
Polyphenol	Gallic acid	573.49, ng/mg
	Xanthophyll	45.64, ng/mg
	Quercetin 3-β-D-glucoside	26.86, ng/mg
	Rutin	21.02, ng/mg
	Trans-Ferulic acid	11.00, ng/mg
	Caffeic acid	9.50, ng/mg
	P-Hydroxycinnamic Acid	7.83, ng/mg
	3,4-Dihydroxybenzoic acid	7.35, ng/mg
	Catechin	5.87, ng/mg
	Apigenin	5.83, ng/mg
	Kaempferol-3-O-glucoside	3.57, ng/mg
	Luteolin	3.37, ng/mg
	Quercetin	2.66, ng/mg
	Vanillin	2.41, ng/mg

## Materials and methods

### Trial duration and location

The trial was conducted at Beijing Sanyuan Green Lotus Dairy Cattle Breeding Center from September to November 2024, lasting 66 days, including a 6-day pre-trial period and a 60-day main trial period.

### Trial materials

*Euphorbia humifusa* extract (EHE), a brown powder, was prepared by extracting the dried whole plants of Earthgrass. A targeted metabolomics analytical approach was established using GC–MS, LC–MS, and complementary detection platforms, enabling the quantification of relative or absolute concentrations of target metabolites through bioinformatics-informed data analysis. Standard calibration curves were constructed to facilitate systematic qualitative and quantitative characterization of targeted metabolites in biological samples, ensuring rigorous analytical validation and reproducibility. Specific components are listed in Table 1.

Milk replacer (MR), was provided by the Beijing Precision Animal Nutrition Research Center.

### Trial animals and experimental design

A total of 48 clinically healthy Holstein female calves (2–3 days old) were selected for the trial. These calves all come from the same pasture and were born within a week. Calves were randomly assigned to four treatments (12 calves/treatment), fed twice daily with MR supplemented with 0 mg (control, CON), 400 mg (group A), 800 mg (group B), or 1,200 mg (group C) of EHE. The specific feeding pattern is listed in Table 2. Calves were separated from their dams immediately after birth and received 4 L of colostrum via gavage within 1 h of birth, followed by a second gavage within 24 h. Following standard operating procedures on experimental dairy farms, Immunoglobulin G (IgG) concentration in colostrum was determined using a handheld Brix refractometer (RHB-32, ATC, Inc., CA, USA; readings >22% Brix) (12). Subsequently, within 72 h postpartum, serum total protein concentration in calves was assessed using a separate handheld Brix refractometer (HT312, ATC, Inc., CA, USA; range: 0–12 g/dL). All experimental calves exhibited serum total protein concentrations >5.5 g/dL, meeting the established adequacy criterion (13). Calves were moved from the calving pen to experimental hutches at 3 days of age. From days 3 to 6, calves were fed a mixture (143 g/L) of on-farm fresh milk and trial MR twice daily (2 L per feeding), with MR inclusion increasing by 1/9 per feeding until full transition to MR by day 7. Nutritional compositions of MR and starter feed are shown in Table 3.

**Table 2:** Experimental design.

Groups	Diets	
	3–30 days of age	30–60 days of age
CON	MR (0 mg)	MR (0 mg)
Group A	MR (400 mg)	MR (800 mg)
Group B	MR (800 mg)	MR (1,200 mg)
Group C	MR (1,200 mg)	MR (2,400 mg)

**Table 3:** Nutrient levels of MR and starter (air-dry basis).

Items^1^	MR	Starter
Ingredient, %		
Corn	–	40.0
Soybean meal	–	25.0
Wheat bran	–	15.0
Expanded soybean	–	10.0
Wheat middlings	–	8.6
CaHPO_4_	–	0.5
Nacl	–	0.9
Nutrients		
CP, %	23.58	22.58
EE, %	15.99	5.03
Ash,%	5.02	3.73
NDF, %	3.98	16.09
ADF, %	2.01	7.07
Ca, %	0.85	0.82
P, %	0.54	0.56
GE, MJ/kg	17.15	15.35

1 The premix provided the following per kg of the starter: ① VA 11000 IU, VD 3000 IU, VE 50 IU, Fe 84 mg, Cu 11 mg, Mn 40 mg, Zn 100 mg, Se 0.3 mg, I 0.8 mg, Co 0.4 mg. ② Nutrient levels were measured values.

### Sample collection and measurements

*Growth performance*: Calves were weighed before morning feeding at the beginning of the experiment and on the 30th, 60th of day of the experiment, and body measurements (withers height, heart girth, body length, and pinbone width) were recorded. Daily feed allocation and leftovers of MR and starter feed were recorded to calculate daily feed intake from the previous day. Total dry matter intake (DMI), average daily gain (ADG), and feed conversion ratio were calculated accordingly.

MR and starter feed samples were collected every 2 weeks and transported to the laboratory for proximate nutrient analysis at the end of the trial. Dry matter content was determined following methods in Feed Analysis and Feed Quality Testing Technology; crude protein (method 990.03), crude fat (method 2003.05), crude ash (method 942.05), calcium (method 985.01), and phosphorus (method 985.01) were measured using AOAC protocols (14). Neutral detergent fiber (NDF) and acid detergent fiber (ADF) were analyzed using the Van Soest method with *α*-amylase treatment (15).

*Serum sample collection and analysis*: On the morning of the 30th and 56th day of the experiment, 10 mL of serum was collected through the jugular vein 2 h after feeding, and immediately centrifuged at 2,000 × g 4 °C for 20 min in a blood collection tube without heparin sodium. The supernatant was divided into 1.5 mL centrifuge tubes and stored at −20 °C for future use to determine the serum’s antioxidant, anti-inflammatory, and immune functions. The determination of serum superoxide dismutase (SOD), malondialdehyde (MDA), catalase (CAT), and glutathione peroxidase (GSH Px) was carried out using a kit colorimetric method (Nanjing Jiancheng Biotechnology Co., Ltd., China). A001 was used to determine SOD, WST-1 method, intra batch CV = 5.50%, inter batch CV = 3.32%; A003 is used to determine MDA, TBA method, intra batch CV = 2.30%, inter batch CV = 5.34%; A007 is used to determine CAT, UV method, intra batch CV = 2.21%, inter batch CV = 6.78%; A005 is used for the determination of GSH Px by colorimetric method, with intra batch CV = 3.56% and inter batch CV = 6.80%. The serum growth hormone (GH), insulin-like growth factor 1 (IGF-1), immunoglobulin IgA, IgG, and IgM, diamine oxidase (DAO), and D-lactate (D-LA) were measured using a bovine ELISA kit (Beijing Laibo Tairui Technology Development Co., Ltd.).

### Network pharmacology analysis

*Target collection*: Using the Traditional Chinese Medicine Systems Pharmacology Database (TCMSP), effective compounds of EHE and their corresponding targets were retrieved based on screening criteria of oral bioavailability (OB) ≥ 30% and drug-likeness (DL) ≥ 0.18. Target proteins were converted to standardized gene identifiers in the UniProt database.^1^ Antioxidant-related targets were searched in OMIM, GeneCards, PharmGKB, and TTD databases using “oxidative stress” as the keyword. After deduplication, intersecting targets were identified, and an EHE-oxidative stress Venn diagram was constructed.

*Construction of EHE-antioxidant target gene network*: Intersection targets from 1.5.1 were subjected to protein–protein interaction (PPI) analysis using the String database. Results were imported into Cytoscape 3.10.3 for topological analysis to construct a visual interaction network of EHE active components and antioxidant targets, as well as identify key antioxidant targets of EHE.

*GO functional and KEGG pathway enrichment analyses*: GO functional enrichment and KEGG pathway analyses of EHE antioxidant targets were performed using R software or the Bioinformatics Platform.^2^

### Statistical analysis

All data were screened for normality using the UNIVARIATE procedure of SAS version 9.4 (SAS Inst. Inc., Cary, NC) and processed for outliers based on the absolute studentized residual value > 3. The growth performance and blood parameters all followed normal distribution.

The ADG, DMI, F: G ratio, serum parameters (GH, IGF-1, IgG, IgA, IgM, DAO, D-LA, SOD, MDA, GSH-Px, CAT), The contrast statement of SAS was used to test the linear, and quadratic effects. The statistical model for repeatedly measured data is as follows:


Yijk=μ+Ti+Dj+TDij+Ck+eijk

where Yjik is the dependent variable, μ is the overall mean, Ti is the effect of ith treatment, Dj is the effect of jth age, TDij is the interaction between treatment and age, Ck is the random effect of kth calf within treatment, and eijk is the residual error. The ADG data using the MIXED procedure with repeated measures of starter intake as a covariate, according to the model as follows:


Yijklm=μ+Ti+Dj+TDij+Ck+β(Xl−X)+eijklm

where Yijklm is the dependent variable, μ is the overall mean, Ti is the effect of ith treatment, Dj is the effect of jth age, TDij is the interaction between treatment and age, Ck is the random effect of kth calf within treatment, β(Xl-X)is the covariate variable, where β is the regression coefficient relating starter intake to ADG, and eijkl is the residual error. The variance components (type VC) covariance structure was used for these repeatedly measured data based on the lowest value for Schwart’s Bayesian Criterion (BIC). The means and standard error of mean data were reported using least squares means and PDIFF.

Body measurements, including wither height, body length, heart girth, hip height, and hip width, were analyzed using one-way analysis of variance (ANOVA) in SPSS 25.0 (IBM Corp., Armonk, NY, USA). Post-hoc comparisons were performed using Duncan’s multiple range test. Statistical significance was declared at *p* < 0.05.

## Results

### Effects of EHE on growth performance of preweaned calves

Table 4 shows the main growth performance and feed conversion rate of calves. It can be seen that the daily weight gain of calves during the entire experimental period increased with the content of EHE in the milk replacer powder. Among them, the daily weight gain of calves in group C was 104 grams/day higher than that of the control group, and the difference reached a significant level between 37 and 66 days of age (*p* < 0.05). Dry matter intake (DMI) was positively correlated with ADG, And there is a time effect (*p* < 0.01) when EHE plays a role, and there is a trend of interaction between age and treatment group (0.05 < *p* < 0.1). Average DMI in group C was significantly higher than in CON (18.28% increase, *p* < 0.05).

**Table 4:** Effects of EHE on growth performance of preweaned calves.

Items	Groups^1^				SEM	*p*-value			
	CON	Group A	Group B	Group C		T	D	T × D	L
Initial weight, kg	41.5	40.8	43.3	43.1	0.61	0.38			
Final weight, kg	86.5	89.7	91.6	95.6	1.42	0.14			
ADG, g/d									
6–66 days of age	738.3	794.4	793.6	842.3	17.15	0.21	<0.01	0.056	0.145
6–36 days of age	727.1	747.1	797.4	813.5	31.93	0.76			
37–66 days of age	797.2^b^	867.4^ab^	821.4^ab^	888.3^a^	12.78	0.05			
Total DMI, g/d									
6–66 days of age	1117.16^b^	1192.85^ab^	1159.17^b^	1321.40^a^	26.84	0.04	<0.01	0.047	0.176
6–36 days of age	839.13	857.46	861.37	877.42	12.78	0.48			
37–66 days of age	1395.19^b^	1528.24^b^	1456.97^b^	1765.38^a^	22.29	0.01			
F/G									
6–66 days of age	1.54	1.51	1.47	1.58	0.03	0.73	0.09	0.238	0.189
6–36 days of age	1.15	1.16	1.10	1.12	0.03	0.97			
37–66 days of age	1.75	1.76	1.77	1.98	0.04	0.92			

1 There were 3 replicates for each feed material and 3 parallel replicates for each replicate. Different letters in the same column indicate significant differences (*p* < 0.05), while no letters indicate insignificant differences (*p* > 0.05); T is the treatment group, D is the age, T × D = *p*-value of interaction between treatment and age, L = contrast test for Linear trend of treatment.

The body size parameters of calves between different strength groups are shown in Table 5. Although there was no significant difference in observed body size indicators, the body size of calves in the EHE added group was higher than that of the control group, and there was a significant increase in Hip height trend (0.05 < *p* < 0.1).

**Table 5:** Effect of EHE on body size index of preweaned calves.

Items	Groups				SEM	*p*-value
	CON	Group A	Group B	Group C		
Wither height, cm	95.80	95.52	98.45	97.86	4.52	0.26
Body steep length, cm	89.57	91.34	91.91	91.37	4.52	0.11
Hip height, cm	102.36	104.22	105.35	108.13	4.87	0.10
Hip width, cm	28.77	28.87	28.95	30.12	1.25	0.29
Heart girth, cm	106.41	106.93	107.82	111.32	6.14	0.81

Different letters in the same column indicate significant differences (*P* < 0.05), while no letters indicate insignificant differences (*P* > 0.05).

### Effects of EHE on serum immune indices and growth hormone in preweaned calves

Serum concentrations of growth hormone (GH), insulin-like growth factor-1 (IGF-1), immunoglobulins (IgG, IgA, and IgM), diamine oxidase (DAO), and D-lactic acid (D-LA) were measured in calves at 30 and 60 d of age (Table 6). Although numerical differences were observed between the 2 time points, these variations did not reach statistical significance (*p* > 0.05). Throughout the experimental period, calves in group C demonstrated the highest serum GH concentration, showing a 20% increase compared with the CON group (*p* < 0.05). This result is positively correlated with the ADG result and exhibits a time effect. There is a significant interaction between treatment and age (*p* < 0.01).

**Table 6:** Effects of EHE on serum immune indicators and growth hormone in preweaned calves.

Items	Groups^1^				SEM	*p*-value			
	CON	Group A	Group B	Group C		T	D	T × D	L
GH, ng/mL									
6–66 days of age	1.35^b^	1.48^ab^	1.45^ab^	1.62^a^	0.03	0.06	<0.01	<0.01	0.851
6–36 days of age	1.32^b^	1.50^ab^	1.37^b^	1.66^a^	0.04	0.02			
37–66 days of age	1.39	1.45	1.59	1.54	0.05	0.62			
IGF-1, ng/mL									
6–66 days of age	83.00	86.09	78.32	86.20	1.78	0.33	0.060	0.766	0.638
6–36 days of age	86.19	91.86	79.24	91.87	2.17	0.09			
37–66 days of age	79.80	80.32	77.22	80.53	2.61	0.97			
IgG, g/L									
6–66 days of age	9.86	9.94	10.07	10.37	0.16	0.70	0.184	0.975	0.642
6–36 days of age	9.58	9.69	9.77	10.45	0.20	0.46			
37–66 days of age	10.08	10.15	10.44	10.30	0.24	0.96			
IgA, g/L									
6–66 days of age	0.67	0.70	0.71	0.70	0.02	0.91	0.271	0.925	0.378
6–36 days of age	0.69	0.75	0.71	0.75	0.02	0.83			
37–66 days of age	0.66	0.66	0.71	0.66	0.02	0.83			
IgM, g/L									
6–66 days of age	2.36	2.36	2.42	2.64	0.07	0.47	0.238	0.605	0.946
6–36 days of age	2.42	2.41	2.51	2.80	0.10	0.44			
37–66 days of age	2.31	2.32	2.32	2.45	0.11	0.96			
DAO, U/ml									
6–66 days of age	2.60	2.55	2.46	2.53	0.06	0.88	0.465	0.844	0.867
6–36 days of age	2.63	2.65	2.45	2.58	0.08	0.82			
37–66 days of age	2.57	2.48	2.48	2.49	0.08	0.98			
D-LA, umol/L									
6–66 days of age	4.91^a^	4.47^ab^	3.91^b^	4.57^ab^	0.13	0.04	<0.01	0.072	0.768
6–36 days of age	4.95	4.72	4.00	5.24	0.19	0.11			
37–66 days of age	4.88^a^	4.22^ab^	3.78^b^	3.91^b^	0.15	0.03			

1 Each feed material had 3 replicates, with 3 parallel subsamples per replicate. In the same column, data denoted by different letters indicate significant differences (*P* < 0.05), while those without letter annotations are not significantly different (*P* > 0.05). Key notations: T represents the treatment group, D denotes age, T × D is the *P*-value for the interaction between treatment and age, and L indicates the contrast test for the linear trend of the treatment effect.

No significant differences were detected in serum immunoglobulin concentrations (IgG, IgA, or IgM) among treatment groups (*p* > 0.05); however, calves receiving EHE supplementation exhibited numerically greater values for all 3 immunoglobulins relative to the control group.

The concentrations of DAO and D-LA in groups B and C were lower than those in the CON group. At 60 days of age, the concentration of D-LA decreased with increasing EHE dose and was significantly lower than that in the CON group (*p* < 0.05).

### Effects of EHE on serum antioxidant capacity in preweaned calves

As presented in Table 7, serum SOD, GSH-Px, and CAT concentrations in calves exhibited a dose-dependent increase with escalating EHE supplementation across treatment groups during the experimental period, though no significant differences were detected among treatments (*p* > 0.05). Bu the concentration of SOD and CAT had a significant effect of changing with age (*p* < 0.05).

**Table 7:** Effect of EHE on serum antioxidant properties of preweaned calves.

Items	Groups^1^				SEM	*p*-value			
	CON	Group A	Group B	Group C		T	D	T × D	L
SOD, U/mL									
6–66 days of age	95.01^b^	96.90^ab^	96.73^ab^	98.92^a^	0.61	0.24	0.022	0.176	0.942
6–36 days of age	93.60^b^	95.98^ab^	94.08^ab^	98.93^a^	0.87	0.11			
37–66 days of age	96.41	97.68	99.39	98.90	0.79	0.65			
MDA, nmol/mL									
6–66 days of age	2.36	2.47	2.41	2.38	0.04	0.81	0.287	0.422	0.834
6–36 days of age	2.10	2.48	2.46	2.38	0.05	0.25			
37–66 days of age	2.58	2.46	2.33	2.37	0.07	0.65			
GSH-Px, U/mL									
6–66 days of age	41.13	38.64	41.19	46.78	3.20	0.84	0.239	0.683	0.909
6–36 days of age	47.46	46.10	45.97	50.34	5.64	0.99			
37–66 days of age	36.39	33.05	33.22	43.22	2.54	0.48			
CAT, U/mL									
6–66 days of age	8.11	7.69	7.97	8.31	0.13	0.37	0.011	0.083	0.387
6–36 days of age	8.82^a^	7.90^b^	8.18^ab^	8.77^ab^	0.16	0.14			
37–66 days of age	7.39	7.55	7.71	7.84	0.83	0.16			

1 In each column, data labeled with distinct letters signify significant differences at the *P* < 0.05 level, whereas unmarked data indicate non-significance (*P* > 0.05). Regarding key notations: “T” stands for the treatment group, “D” represents age, “T × D” denotes the *P*-value for the interaction between treatment and age, and “L” indicates the contrast test assessing the linear trend of the treatment effect.

Similarly, MDA concentrations did not differ between groups (*p* > 0.05). Notably, calves in group C displayed a 12.5% greater serum SOD concentration compared with the CON group at 30 d of age (*p* < 0.05).

### Network pharmacology analysis

**Figure 1:**
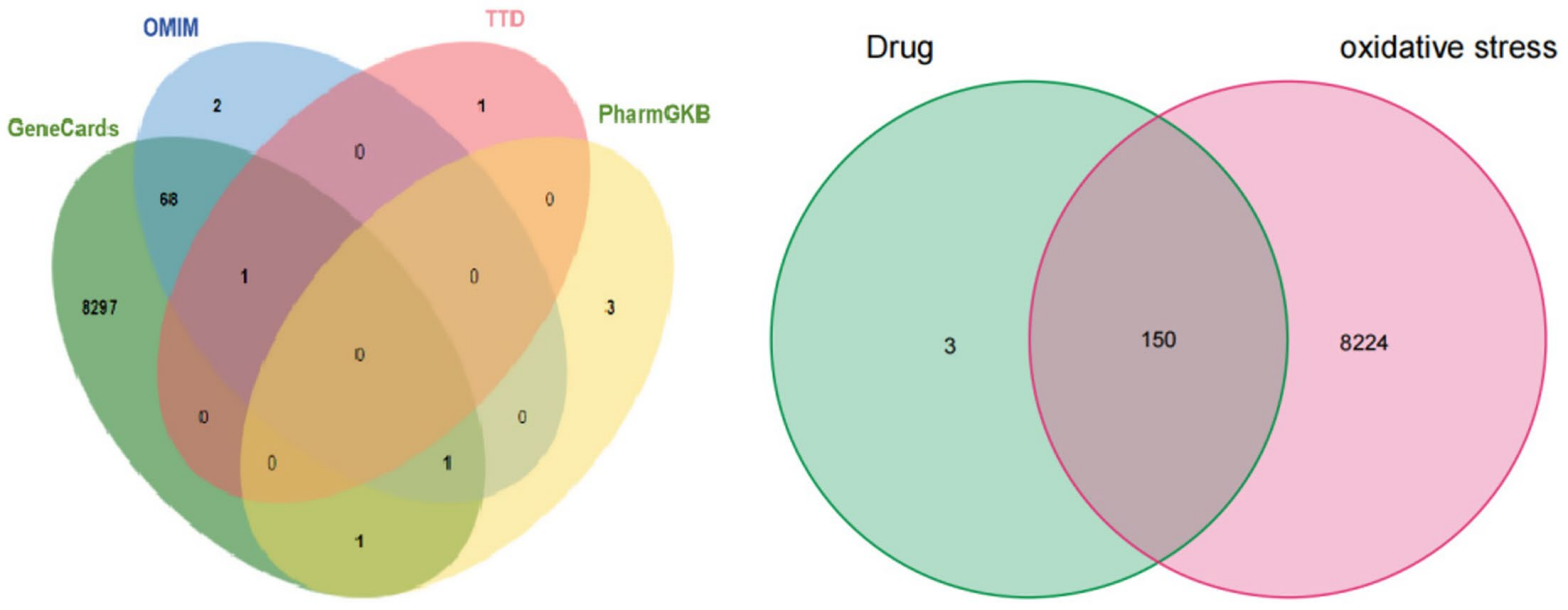
Disease related targets **(A)** and drug disease target intersection **(B)**. **(A)** Venn diagram of inflammation-related targets retrieved from the OMIM, PharmGKB, TTD, and GeneCards databases; **(B)** Venn diagram of the intersection between EHE-related targets (131) and oxidative stress targets (8377), with 150 common targets.

*Acquisition of EHE targets, antioxidant-related targets, and potential action targets*. Based on the TCMSP database, 38 active components were initially obtained, and 13 active components were screened using oral bioavailability (OB) ≥ 30% and drug-likeness (DL) ≥ 0.18 as criteria. Targets of these active components were standardized using validated genes from the Uniprot database, resulting in 20 active components corresponding to 256 action targets; 6 potential targets were identified after deduplication. Using “oxidative stress” as the search term, antioxidant-related targets were retrieved from OMIM, GeneCards, PharmGKB, and TTD databases. Data from each database were processed using the “Venn” package in R for deduplication and intersection analysis, followed by construction of a drug-disease Venn diagram (Figure 1).

**Figure 2:**
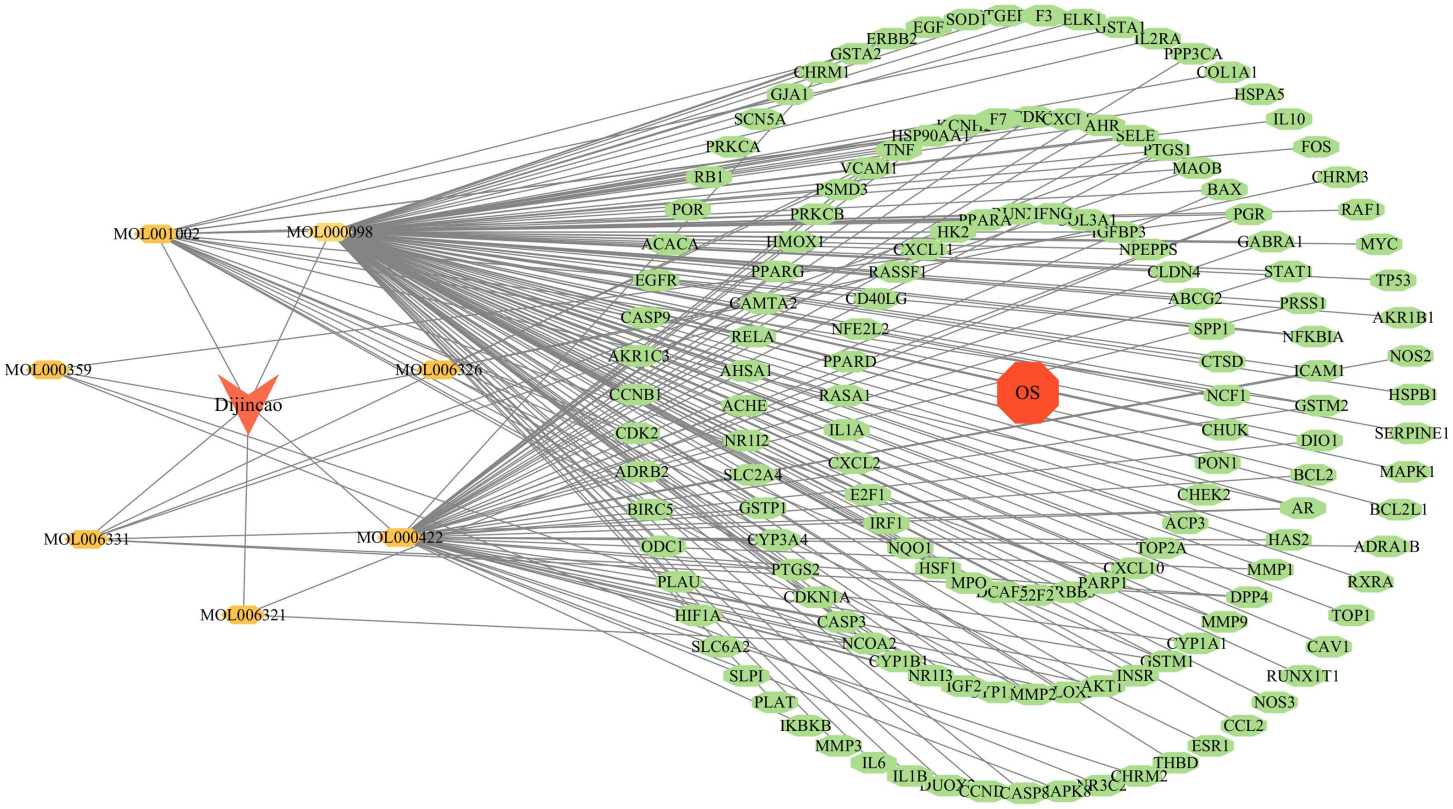
Active ingredients-target plot: EHE active components (orange nodes) and antioxidant targets (yellow nodes); edges represent the interaction between active components and targets. The size of nodes is positively correlated with the number of interactions (degree value), indicating that components with larger nodes and targets with larger nodes play a core role in the network.

*Construction of PPI network and screening of core targets*. Twenty oxidative stress-related active components and 150 associated genes were imported into the Cytoscape database to construct an active component-target network (Figure 2), demonstrating that EHE alleviates oxidative stress through multiple targets and components.

**Figure 3:**
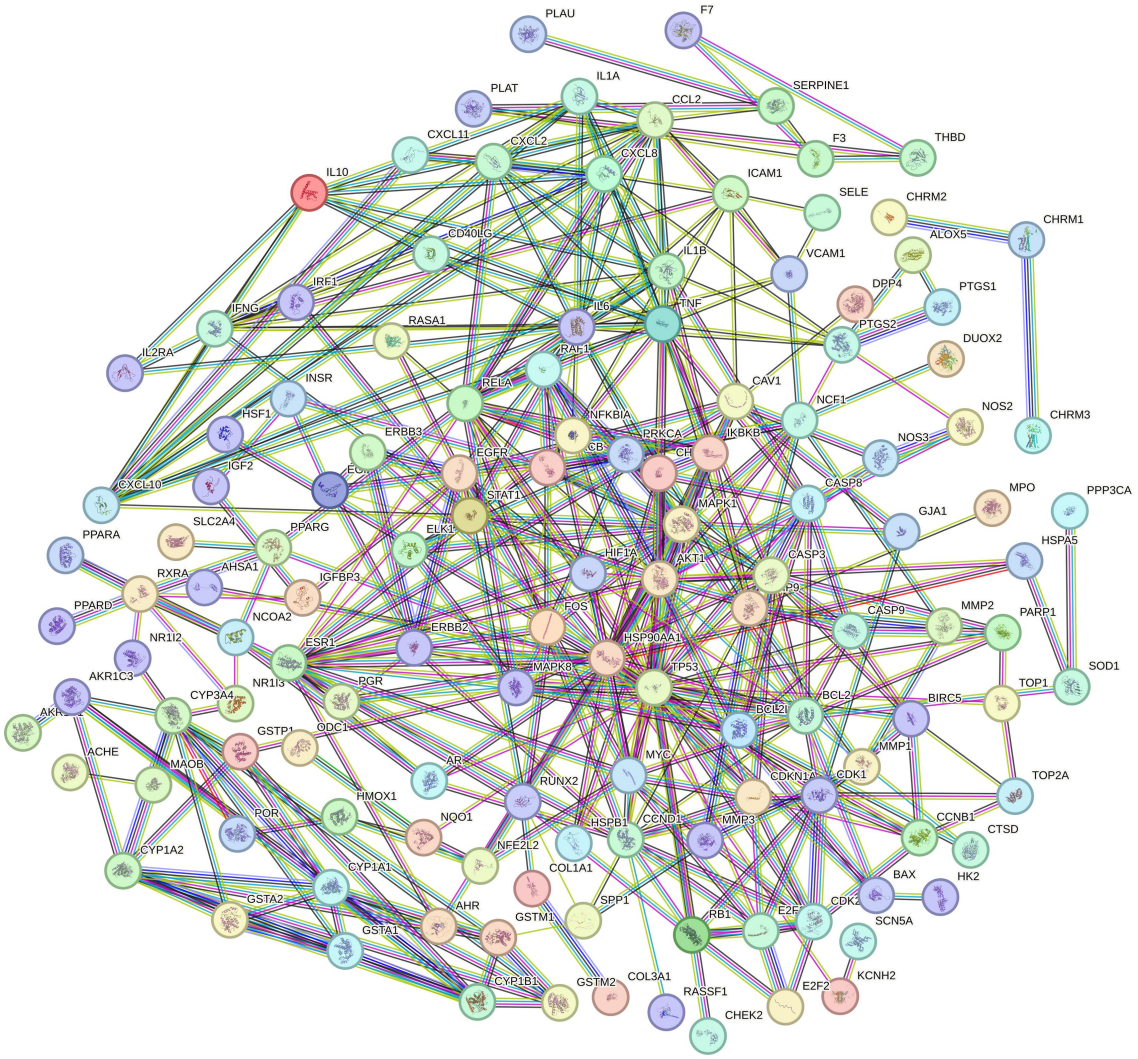
PPI network diagram of EHE protein: nodes represent target proteins; edges represent protein–protein interactions (STRING database, minimum combined score = 0.9). Disconnected nodes are removed. Node color depth is positively correlated with degree value (interaction frequency), and core targets are dark red.

**Figure 4:**
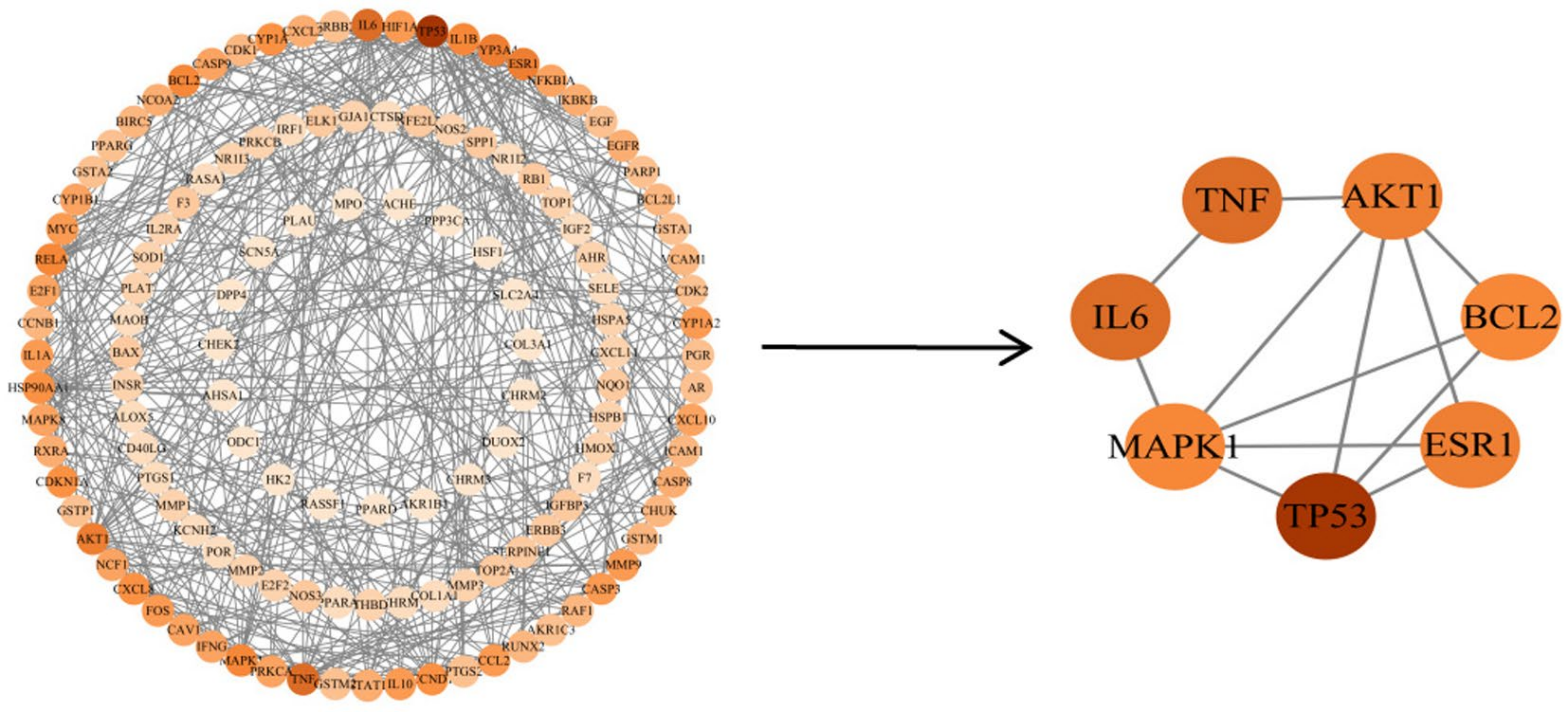
Core target gene network: shown in the diagram are the top 7 core targets (BCL2, IL6, ESR1, MAPK1, AKT1, TNF, TP53), identified via the CytoNCA plugin (Cytoscape 3.10.3). The x-axis represents degree value (protein interaction count), and the y-axis represents betweenness centrality (capacity to mediate inter-node interactions). With high degree and betweenness centrality, these targets play a critical role in the antioxidant network.

Target genes of EHE’s main active components related to oxidative stress were uploaded to the String database, and the filtered active components and disease-critical targets were imported into Cytoscape 3.10.3 to generate a visual interaction map of EHE components and targets. Using a minimum interaction score of 0.9 and removing isolated nodes, a PPI network file was obtained (Figure 3), containing 130 nodes and 834 edges. As shown in Figure 4, the PPI file was analyzed in Cytoscape with the CytoNCA plugin, identifying key targets for EHE-mediated oxidative stress alleviation in calves: ESR1, MAPK1, AKT1, TNF, BCL2, IL6, and TP53.

*GO functional and KEGG pathway enrichment analyses*. GO enrichment analysis of the targets was performed using R software, identifying 151 GO terms for 40 targets. Using a significance threshold of *p* < 0.05, the top 10 most enriched pathways were selected for visualization. Biological processes primarily included response to xenobiotic stimulus, response to lipopolysaccharide, and response to oxidative stress.

Cellular components were mainly associated with membrane raft, membrane microdomain, plasma membrane raft, and caveola. Molecular functions with high enrichment relevance included DNA-binding transcription factor binding, nuclear receptor activity, ligand-activated transcription factor activity, and transcription coregulator binding.

**Figure 5:**
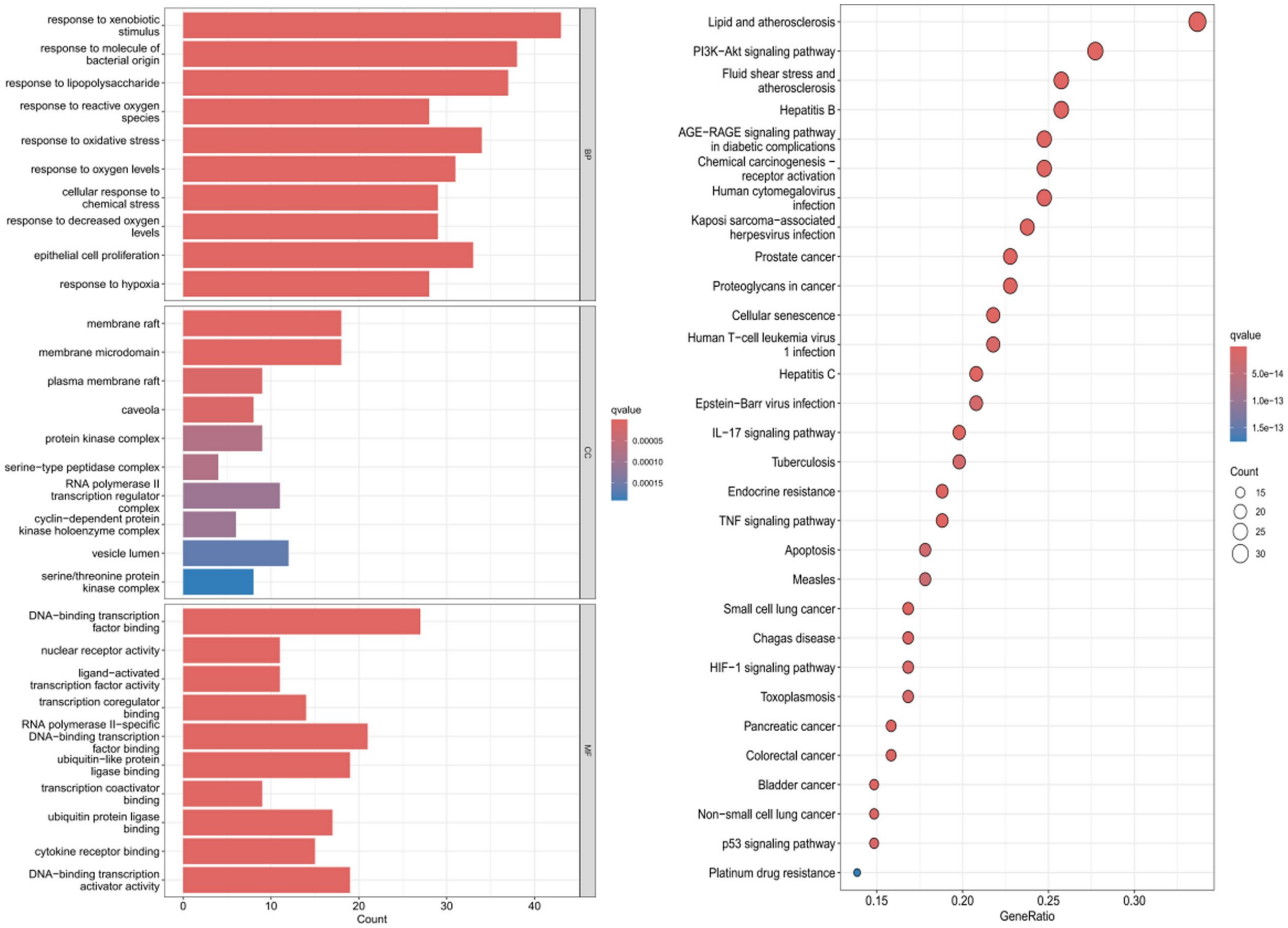
GO **(A)**. KEGG **(B)** enrichment analysis. **(A)** The left panel shows the top 10 enriched biological processes (*p* < 0.05), the right panel shows the top 10 enriched cellular components (*p* < 0.05), The x-axis represents GeneRatio (proportion of target genes in the total genes of the pathway), and the y-axis represents the pathway name. **(B)** The x-axis represents the number of target genes enriched in the pathway, and the y-axis represents the pathway name. The top 10 significantly enriched pathways (*p* < 0.05) are shown.

KEGG pathway screening with *p* < 0.05 identified 30 signaling pathways, including *lipid and atherosclerosis*, *PI3K-Akt signaling, atherosclerosis*, *diabetic complications*, *human cytomegalovirus infection*, *cellular senescence*, and *endocrine disorders*. Relevant results are shown in Figure 5.

## Discussion

### Effects of EHE on feed intake and growth performance in preweaned calves

In modern animal husbandry, the healthy growth of calves is fundamental to ensuring subsequent breeding efficiency. The preweaned period, a critical stage for calf development, imposes strict requirements on nutrient intake and environmental conditions (16). *Euphorbia humifusa* (EHE), a common Chinese natural plant—contains multiple bioactive components. Our trial’s EHE analysis identified main actives: saponins, flavonoids, saccharides, and polyphenols (gallic acid, lutein, quercetin 3-*β*-D-glucoside, rutin, trans-ferulic acid, etc.). As an animal feed additive, these components may improve health, boost growth, and reduce antibiotic reliance, aligning with green farming.

In this study, calf average daily gain (ADG) increased with increasing EHE dosage, and group C had the highest serum growth hormone (GH) concentration, significantly 20% higher than the CON group, which correlated positively with ADG results. This may be related to its multiple bioactive components. Saponins, complex natural organic compounds widely present in plants, exhibit diverse functions. As feed additives in livestock and poultry farming, they improve growth performance, enhance immunity, alleviate stress, and optimize the rearing environment. Li et al. (17) noted 0.01% quillaja saponins (QS) in growing-finishing pig diets improved growth and reduced fecal CO₂. Lu et al. found 45 mg/d Pulsatilla saponin B4 in lactating calf milk replacer enhanced 14–28-day-old calf growth (18). Flavonoids, a class of natural products with broad biological activities, have been shown to enhance calf growth performance (5, 19). For example, baicalin (a major flavonoid in Scutellaria baicalensis) significantly improves growth by increasing intestinal digestive enzyme activities (amylase, protease, lactase) related to protein and starch digestion and stimulating growth hormone release, thereby enhancing daily gain and feed efficiency (5, 19). Xi et al. (20) reported that dietary supplementation with 300 mg/kg total flavonoids from *Euphorbia humifusa* led to a numerical increase in growth performance, brought about a numerical rise in immune function, and resulted in a numerical elevation in antioxidant capacity in Ross 308 broilers. This outcome is consistent with the findings of this study.

Phenolic compounds, defined as aromatic hydrocarbons with hydroxyl group substitutions on the benzene ring, contribute to gastrointestinal development, health, and growth performance in livestock (21). Optimal 300 mg/kg gallic acid (GA) mitigates broiler stress, improves antioxidants, and enhances breast muscle quality (21). In ruminants, Xu et al. (22) showed GA in preweaned calf starter feed improved growth; our EHE’s high GA likely boosted ADG. Wang et al. (6) reported that rutin supplementation increased milk replacer intake and feed conversion ratio in calves, consistent with our observation of higher DMI in group C compared to CON. Ferulic acid (FA), a natural compound with antioxidant and antimicrobial activities, has been proposed as a novel growth promoter for meat-producing animals, improving ADG and carcass weight without altering meat quality parameters (23). As one of the main polyphenolic components in EHE, p-hydroxycinnamic acid acts as a natural nutritional additive to enhance production performance in livestock; dietary supplementation has been shown to stimulate feed intake, accelerate growth, and reduce feed conversion ratio in lambs (24). Collectively, these findings suggest that the diverse bioactive components in *Euphorbia humifusa* act independently and synergistically to promote calf growth.

### The effect of EHE on serum immune function of preweaned calves

The immune protection mechanism of newborn ruminants consists of two major defense modes of the organism against specific pathogens: active immunity and passive immunity. In the early stages of life (0–35 days old) of newborn calves, their own specific immune response system has not yet completed developed, and resistance to pathogenic microorganisms at this stage mainly relies on maternal inherited immunity. The material basis of this protective mechanism comes from the abundant maternal antibodies (IgG, IgM, etc.), immune active cells, and various bioactive components in colostrum.

Throughout the entire trial period, there were no significant differences in serum concentrations of IGF-1, IgG, IgA, and IgM among the groups. However, it can be observed that the experimental group fed with EHE had slightly higher concentrations of IGF-1, IgG, IgA, and IgM compared to the CON group. An increase in D-LA levels in the blood usually reflects an increase in intestinal mucosal permeability. The DAO and D-LA concentrations in Group A, Group B, and Group C calves were numerically lower than those in the CON group, and at 60 days of age, with an increase in EHE feeding, the concentration of D-LA decreased significantly compared to the CON group.

Previous studies by our team have shown (25–27) that flavonoids can enhance the immunity of calves. Yin et al. (28) investigated the effects of licorice flavonoid powder (LEP) on the antioxidant capacity and immunity of weaned piglets, and found that supplementing with 150 mg/kg licorice flavonoid powder in the diet could upregulate IgG content, alleviate immune organ inflammation, and improve the immune function of piglets. Yin et al. (28) found that flavonoids can significantly increase IgA and IgG levels. In addition, some differential bacterial genera are highly correlated with serum cytokine and immunoglobulin levels, maintaining intestinal homeostasis and relieving diarrhea. Adding 500 mg/kg rutin to the diet can increase serum IgA levels and reduce TNF - *α* levels, which may be related to enhancing immunity by inhibiting the NF-κB signaling pathway and increasing intestinal antioxidant capacity by activating the Nrf2/HO-1 pathway.

Superoxide dismutase (SOD), glutathione peroxidase (GSH-Px), catalase (CAT), and malondialdehyde (MDA) are substances closely associated with redox reactions in organisms. As a metalloenzyme, SOD scavenges superoxide anion radicals; as a selenium-containing enzyme, GSH-Px catalyzes peroxide reduction; and as an iron-porphyrin enzyme, CAT decomposes hydrogen peroxide. Together, they form the body’s antioxidant defense system, maintaining intracellular redox balance and protecting cells from oxidative damage. Studies have shown that water-soluble powder of EHE increases the production of antioxidant enzymes in porcine intestinal epithelial cells, exerting antioxidant effects (29). In this study, EHE supplementation significantly increased serum SOD concentration and decreased CAT concentration in calves, which may be related to the antioxidant properties of its flavonoid and polyphenolic components.

Zeng et al. (30) reported *in vitro* that flavonoids restored d-galactose-induced decreases in hepatic GSH-Px1, CAT, SOD1, and SOD2 mRNA expression to varying degrees, effectively increasing SOD, CAT, and GSH-Px concentrations. Tu et al. (31) found that dietary supplementation with 500 mg/kg flavonoids significantly reduced diamine oxidase (DAO) activity in jejunal mucosa, increased CAT, total antioxidant capacity, and GSH-Px activity, enhanced SOD activity and glutathione (GSH) content in ileal mucosa, and significantly decreased MDA content in ileal mucosa (31, 32). Kaempferol, a naturally occurring flavanol and potent antioxidant, shows potential as a functional agent for intestinal health (33). Hu et al. (5) demonstrated that low-dose baicalin supplementation significantly increased serum SOD, CAT, and GSH-Px concentrations while decreasing MDA concentration, consistent with our findings. Interestingly, EHE exhibited stronger antioxidant effects at 30 days of age, possibly because calves face birth, colostrum deprivation, and environmental stress within the first 30 days of life. Research suggests that EHE is more beneficial for calves under disease or high stress, explaining their improved antioxidant status in the first few weeks after birth.

Immunity and growth complement each other and are inseparable. The above studies indirectly demonstrate that EHE can enhance the immune system of preweaned calves, which is beneficial for their healthy growth.

### Mechanism of EHE in alleviating oxidative stress based on network pharmacology

In order to analyze the mechanism of EHE antioxidant stress, this study combined animal feeding experiments with network pharmacology to construct an effective ingredient serum antioxidant index antioxidant target network. The results of this experiment indicate that: The active compounds in EHE mainly exert their effects through key targets such as IL6, TP53, MAPK1, AKT1, TNF, BCL2, ESR1, etc. They may exert antioxidant effects through various pathways such as participating in cellular redox reactions and regulating cell proliferation (34).

Zhu et al. (35) demonstrated that flavonoids reduce endothelial cell damage by decreasing IL6 expression, thereby mitigating apoptosis, oxidative stress, and inflammation. It may be that IL6 can induce the synthesis of acute antioxidant proteins in the liver, which can promote the differentiation of B cells and antibody secretion, enhance the body’s immune defense ability, and reduce inflammation and oxidative stress caused by infection. Meanwhile, IL6 may also regulate the activity of immune cells such as macrophages to produce an appropriate amount of ROS for sterilization and other immune processes, avoiding excessive ROS production and oxidative damage.

BCL2 regulates calcium (Ca^2+^) signaling to promote cell proliferation and enhance resistance to apoptosis (35). TP53-induced glycolysis and apoptosis regulator (TIGAR) is a key gene encoding glycolytic enzymes and regulating ROS expression (36). It activates antioxidant gene transcription while inhibiting pro-oxidative genes, such as glucose-regulated protein 78 (GRP78), to reduce endoplasmic reticulum stress–induced ROS production (37). Moreover, TP53 maintains its stability and activity by inhibiting MDM2 expression, lifting MDM2-mediated negative feedback to sustain antioxidant and tumor-suppressive functions (38).

Mitogen-activated protein kinase 1 (MAPK1) activates antioxidant transcription factors to regulate intracellular redox status. For example, it maintains intracellular glutathione levels by modulating the activity of glutathione metabolism-related enzymes. As a key intracellular antioxidant, glutathione scavenges free radicals and protects cells from oxidative damage (39, 40). GO and KEGG enrichment analyses of key targets revealed that oxidative stress is a convergent factor in diseases like cancer and liver injury, and mitigating oxidative damage is a common strategy for treating related conditions (41, 42).

Network pharmacology further demonstrates that EHE alleviates oxidative stress through multiple mechanisms, including immunosuppression, anti-inflammation, antiviral, and antitumor effects. Thus, EHE exhibits a multi-component, multi-target, multi-pathway mode of action in calves. The diverse components of EHE may act through multiple targets to exert anticancer and liver-protective effects, suggesting promising applications in animal production.

## Conclusion

Based on the experimental findings, the following conclusions were drawn:

1 Growth performance parametersSupplementation of *Euphorbia humifusa* extract (EHE) in milk replacer enhanced growth performance, improved antioxidant capacity, and strengthened immune function in preweaning calves. These effects became more pronounced with prolonged feeding duration.

2 Antioxidant mechanismNetwork pharmacology revealed that EHE exhibits multi-component, multi-target, and multi-pathway characteristics. Its antioxidant effects are mediated through interactions with key targets (e.g., IL6, TP53, MAPK1, AKT1, TNF, BCL2, and ESR1), modulating signaling pathways associated with immune response and apoptosis.

## Data Availability

The original contributions presented in the study are included in the article/supplementary material, further inquiries can be directed to the corresponding author.

## Footnotes

^1^ https://www.uniprot.org

^2^ https://www.bioinformatics.com.cn/

## Glossary

LBE: Licorice-Bitter Wood Extracts
AMP: Antimicrobial peptides
MR: Milk replacer
ADG: Average daily gain
DM: Dry matter
CP: Crude protein
EE: Ether extract
NDF: Neutral detergent fiber
ADF: Acid detergent fiber
Ca: Calcium
P: Phosphorus
BW: Body weight
ADFI: Average daily feed intake
ADG: Average daily gain
FCR: Feed conversion ratio
KEGG: Kyoto Encyclopedia of Genes and Genomes
CAZy: Carbohydrate-active enzymes
AA: Activity
CBM: Carbohydrate binding modules
CE: Carbohydrate esterases
GH: Glycoside hydrolases
GT: Glycosyl transferases
PL: Polysaccharide lyases
