# Correction: Effects of *Euphorbia humifusa* extract on growth performance and serum biomarkers in preweaned calves

**DOI:** 10.3389/fvets.2025.1755580

**Published:** 2026-01-23

**Authors:** Chuntao Zhang, Zhongying Xing, Guishan Xu, Yan Tu, Qiyu Diao

**Affiliations:** 1 Key Laboratory of Feed Biotechnology of Ministry of Agriculture and Rural Affairs, Feed Research Institute of Chinese Academy of Agricultural Sciences, Beijing, China; 2 Animal Science and Technology College, Tarim University, Alar, China

**Keywords:** antioxidant, calves growth, *Euphorbia humifusa* extract, milk replacer, network pharmacology, plant extract

An incorrect **Funding** statement was provided. The correct funding statement reads: “The author(s) declared that financial support was received for this work and/or its publication. This research was funded by Prediction model for methane emission reduction in ruminants and emission reduction technology based on metagenomics (2016YFE0109000).

The original version of this article has been updated.

